# Polymer Systems with Correlated Activity: Stars Versus Linear Chains

**DOI:** 10.3390/molecules30224442

**Published:** 2025-11-17

**Authors:** Aleksandr I. Buglakov, Prabha Chuphal, Vladimir Yu. Rudyak, Alexander V. Chertovich, Vladimir V. Palyulin

**Affiliations:** 1 Semenov Federal Research Center for Chemical Physics, Kosygina, 4, 119991 Moscow, Russia; chertov@polly.phys.msu.ru; 2 Faculty of Physics, Lomonosov Moscow State University, Leninskie Gory 1, 119991 Moscow, Russia; 3 Independent Researcher, 119991 Moscow, Russia; 4 School of Physics and Astronomy, Tel Aviv University, Tel Aviv 69978, Israel

**Keywords:** active polymers, star polymers, active matter, gyration radius, active Brownian particle, correlated activity, stretching

## Abstract

Using molecular dynamics simulations, we explore the impact of correlated monomer activity and star topology on the structure and dynamics of active polymers. Unlike uncorrelated active Brownian particle (ABP) stars, correlated activity induces a rather steep stretching of the star polymer at intermediate activity levels. This stretching is characterized by transitions between distinct, metastable states defined by the coordinated movement of the arms, leading to novel collective dynamics. The behavior is consistent with experimental observations of active oligomers, highlighting the critical role of activity correlations for the understanding and modeling of active polymers.

## 1. Introduction

Systems that perform motion with energy-consuming elements are known as active matter. Their dynamics essentially occur in non-equilibrium, which results in markedly different behavior compared to their equilibrated counterparts [1]. The study of active matter has opened new avenues for the understanding of various natural phenomena and has provided a means to programme new ways of control and self-assembly [2,3,4]. Active matter systems span a wide range of scales from the microscopic to the macroscopic world, from self-propulsion in a directed fashion by anchored actin and microtubules over the carpet of motor proteins [5,6,7,8,9], an active cytoskeleton of eukaryotic cells [10], to the collective behavior of groups of animals and bird flocks [11,12].

Most of the up-to-date research utilizes models with active agents having a small number of uncorrelated forces, such as colloidal Janus particles [13,14]. However, more complex systems such as active polymers possess rich and often counter-intuitive dynamics [15,16,17,18,19,20], as is anticipated in theoretical [8,19,21,22] and simulation [15,16,17,19,23,24,25,26] studies. In experiments, active polymer-like systems could be produced as chains of colloidal particles [27,28,29] or even living beings such as thin *Tubifex tubifex* worms [30,31]. Biopolymers often operate in an active environment or have intrinsic activity. For instance, active microtubules, or actin filaments, are responsible for the dynamics of cytoskeleton and chromatin [32]. Altogether, the behavior of active polymers is determined by the interplay of activity and restrictions on degrees of freedom due to chain connectivity [22].

Activity of the polymer beads or segments is modeled by a few distinct approaches. The first analytical theories used the Rouse model for flexible [33] and semiflexible chains [7,8,34] with single segments being active Brownian particles (ABPs), i.e., the activity was essentially uncorrelated. Some simulation papers also use ABP activity for segments [16]. While the ABP model can represent an implicit account of the active medium interacting with a passive polymer [35,36], it fails to catch the inherently occurring correlations between the active segments. In analytical studies, correlations can be introduced through an active force tangential to the chain as in the polar model [37]. Simulations permit a larger set of correlated activity models for the exploration. Apart from the polar/tangential model [18,38,39,40], the collective activity in active polymers was simulated with the Vicsek model [23,41] and the velocity-aligned model [25]. One of the main differences between the correlated activity models is in the correlation range. Under more or less typical parameters, the Vicsek model produces almost fully correlated movement, i.e., the correlations are effectively long-ranged, which is hardly the case in experiments. The relevant experimental realizations have rather medium-range correlations of activity. These can be generated, for instance, by the velocity-aligned model.

**Figure 1:**
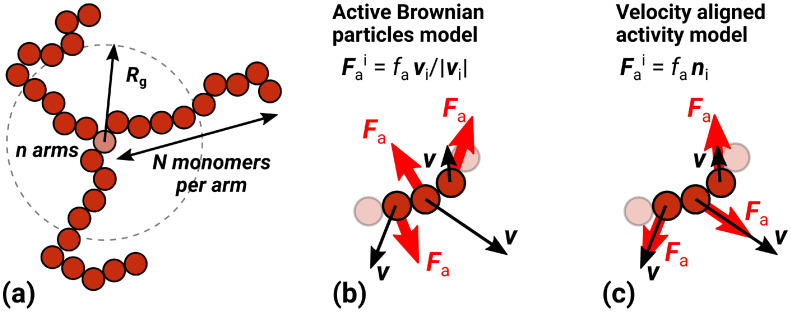
(**a**) An example of a star polymer with a number of arms n=3 and a number of beads per arm N=10. (**b**) Active Brownian particle model. Individual active forces (red arrows, $F_{a}$) and particle velocities (black arrows, v) are uncorrelated. (**c**) Velocity aligned activity model. Active forces act along particle velocities.

In conventional polymer systems, many non-trivial effects are produced by a departure from the linear architecture of polymer molecules or by the change in chemical composition. The latter leads to effects such as phase separation and micelle formation. The former is also of great interest because, in some applications, the branched structures (stars, combs, grafts, cross-linked, etc.) [42,43,44,45,46,47] have advantages over linear polymers because of their tunable parameters. The star architecture is the simplest example of non-trivial connectivity in polymer systems (Figure 1a). Star topology introduces a new kind of correlations in polymer molecules, at least around the star center. This correlation appears due to both the connectivity and the increase in a local density of monomers, i.e., the growth of the excluded volume (crowding) effects. For passive star polymers, the problem of influence of the number of the arms and their length on the shape and the size of the molecule was recently revisited in [48], both with analytical theory and DPD simulations. The authors addressed discrepancies in previous theoretical [49,50,51,52] and modelling [53,54,55] scalings. The dynamics star-shaped crowders is interesting in the case of an interplay between the excluded volume, configurational flexibility, and a weak attraction that enhances the lateral diffusion [56]. Star-like structures are also used to generate a viscoelastic polymer environment for investigating the diffusion of active Brownian particles [57]. The passive star polymers have a wide range of applications from the synthesis of nanomaterials [58] and nanotechnology [59,60] to drug or gene delivery [42,61] and bioengineering [58].

To the best of our knowledge, there are no studies of the interplay of polymer branched topology and correlated/uncorrelated activity. Hence, in this paper, we explore the interplay of both correlated and uncorrelated activity with a star polymer topology (Figure 1a) and compare the findings with known results of the passive and linear counterparts. Among the existing activity models, we choose ABP and the velocity-aligned models as representatives of both isotropic activity and a correlated one. We find that the branched topology plays a significant role only if activity is correlated.

## 2. Results

We modeled active star polymers as a set of *n* individual arms of *N* monomer units each, attached to the central monomer (Figure 1a). The number of arms *n* varied from 2 to 6. In the case of two arms, the star becomes a linear chain. The arm length *N* varied between 10 and 100.

We used an underdamped Langevin dynamics to study two different models of monomer activity: the Active Brownian particles model and the velocity-aligned activity model (the detailed description is provided in Section 3). In both cases, all beads were considered active and therefore pushed by active forces $F_{a}^{i}$, but the resulting dynamics of the active forces was different (see Figure 1b,c). The velocity-aligned activity model, studied recently in [25], takes the correlations of active forces into account as follows. An active force drives a particle along its own velocity, which is directly affected by interactions with neighbor particles. For example, this mechanism can be implemented in systems with activity caused by the local surface tension gradient. The deformation of the particles determines their activity, and during collisions, it leads to changes in the active force direction. We neglect the interaction time and assume that this interaction and the change in the direction of the active force occur instantaneously, leading to an effective correlation of the bead velocities.

**Figure 2:**
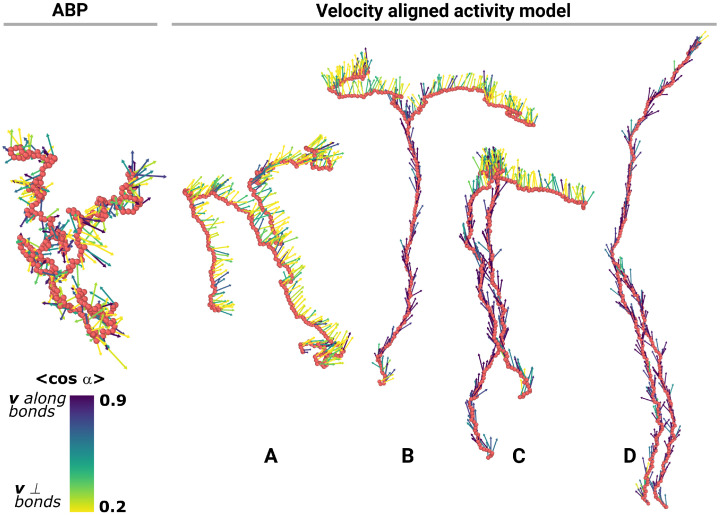
Instant snapshots of a typical active polymer star steady states with n=3 arms at high activity within two different activity models. Monomers are shown as red spheres. Arrows represent monomer velocities v colored by the correlation between velocity and local bonds direction <cosα>. Active Brownian particle (ABP) stars typically remain in a low-correlated steady state (**left**). Velocity aligned activity model stars reveal a set of steady states with either parallel or perpendicular ordering of each arm. Here, n=3 arms lead to the existence of four states, (**A**–**D**).

Let us start with the visual inspection of occurring steady states. Obviously, both models produce the same thermal equilibrium at zero monomer activity. However, as the activity grows, the resulting steady states become strikingly different (Figure 2). In the ABP model, we observe gradual swelling as activity grows, for any number of arms *n*, including a linear polymer (n=2). For the velocity aligned model, all systems remain disordered below a critical activity $f_{a}/\left(k_{B}T\right)\approx 100$. Above it, linear polymers tend to order into one of the two states, where the velocities of all monomers almost coincide, thus giving the center of mass a solid velocity. In the first state, the polymer becomes almost two-dimensional, with the velocities oriented perpendicular to this 2D plane and to the bonds (the state is described in detail in [25]). In the second state, the polymer elongates, and the velocities of monomers are directed along the direction of the polymer. In the case of stars (n>2), the ordering occurs after the same activity threshold $f_{a}/\left(k_{B}T\right)\approx 100$. However, in this case, each arm of the star chooses between these two states independently, thus forming multiple possible steady states for the system (see Figure 2). Below, we study this peculiarity in more detail and characterize it with static and dynamic order parameters.

### 2.1. Structure of Stars Made of Velocity Aligned Active Particles

**Figure 3:**
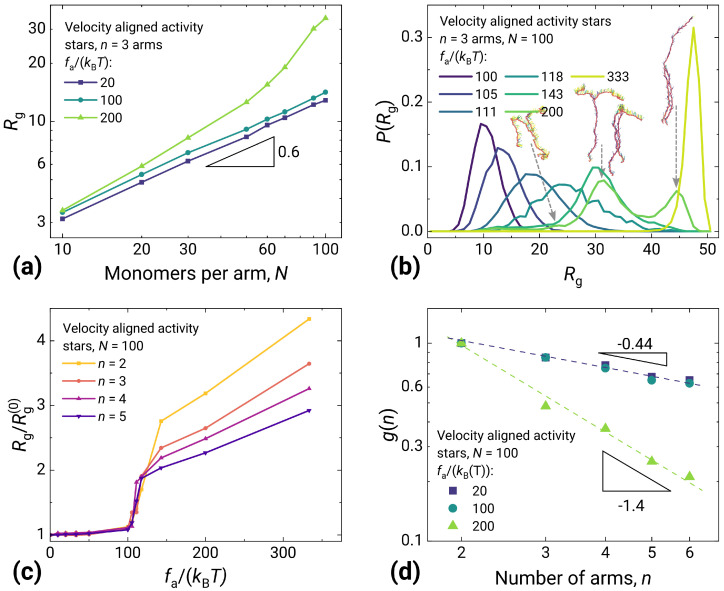
Characteristics of active stars made of velocity aligned active monomers. (**a**) Radius of gyration $R_{g}$ dependency on arm length *N*. Below $f_{a}/\left(k_{B}T\right)\approx 100$, scaling remains close to 0.6. At higher activities, the system exhibits more swollen conformations. (**b**) Distribution of $R_{g}$ for various activities $f_{a}/\left(k_{B}T\right)$, in the case of n=3, N=100. In the range $100≲f_{a}/\left(k_{B}T\right)≲120$, the shift in the distribution similar to ABP stars (see below) is observed. At higher activities, $R_{g}$ shows multimodal distribution, associated with multiple states of the system. (**c**) Swelling ratio $R_{g}/R_{g}^{\left(0\right)}$ as a function of activity $f_{a}/\left(k_{B}T\right)$, for various numbers of arms *n* and N=100. A sharp transition from a disordered to ordered regime is obtained between $f_{a}/\left(k_{B}T\right)\approx 100$ and ≈120. (**d**) Parameter g(n) of N=100 stars with different numbers of arms *n* scales as −0.6 in the disordered regime and close to −1.5 in the ordered regime.

From the snapshots, it is clear that the velocity-aligned model produces non-trivial results. To quantify them, we first show the dependence of the scaling of gyration radius on the length of an arm *N* (Figure 3a) for the star polymer with n=3. While at high temperatures the scaling corresponds to the classical excluded volume exponent ≈3/5, after the chain stretches we see a different behavior (the upper, green curve). At longer lengths of arms, the exponent is higher. The gyration radius for linear polymers (n=2) and stars with n=3,4,5 remains almost constant for $f_{a}/\left(k_{B}T\right)$ below the abrupt transition at ∼100 when the radius grows rapidly (Figure 3c). Above the transition, the overall stretching continues but at a substantially slower pace. Both the amplitude of a transition and the steepness of the post-transition stretching decrease with *n* due to a larger monomer crowding at higher *n*. Although the obtained transition is not point-sharp due to single molecule simulations, we consider this a phase transition, based on the evidence of static order parameters and transition kinetics for linear polymers studied in [25].

The distribution of gyration radii at different activities for the three-arm star gives a more detailed picture (Figure 3b). While during the transition (the curves for $f_{a}/\left(k_{B}T\right)$ between ∼100 and ∼120), the system only swells, after the transition (the curve for $f_{a}/\left(k_{B}T\right)=143$) it clearly demonstrates multistability. At a further increase of activity, the more prolonged states gradually become more populated. Finally, for $f_{a}/\left(k_{B}T\right)≳300$, only the most stretched state remains populated, in which the whole system is stretched in a line and moves along this line.

**Figure 4:**
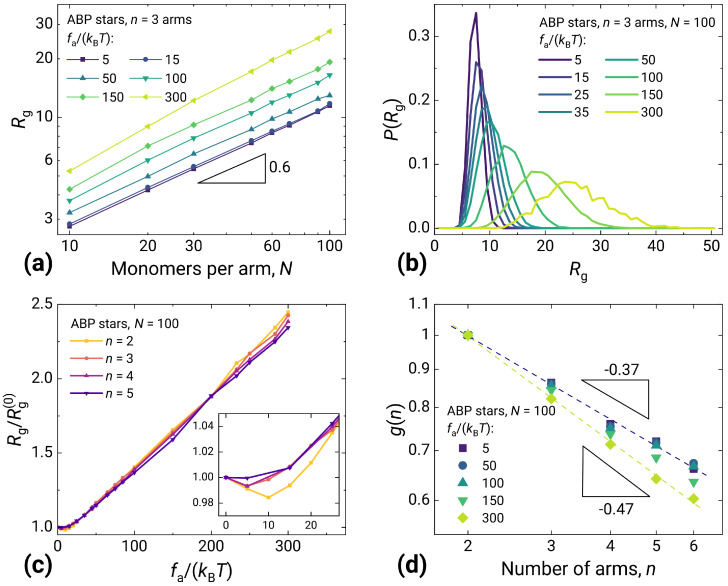
Characteristics of active stars made of ABP monomers. (**a**) Radius of gyration $R_{g}$ dependency on arm length *N*. For all monomer activities $f_{a}/\left(k_{B}T\right)$, scaling remains close to 0.6. (**b**) Distribution of $R_{g}$ for gradually shifting at increasing activity $f_{a}/\left(k_{B}T\right)$, in the case of n=3, N=100. (**c**) Swelling ratio $R_{g}/R_{g}^{\left(0\right)}$ as a function of activity $f_{a}/\left(k_{B}T\right)$, for various numbers of arms *n* and N=100. Linear growth observed after the initial collapse/plateau. (**d**) Parameter g(n) of N=50 stars with different numbers of arms *n* scales between −0.35 and −0.5 in the studied range of activities.

The presence of multiple steady states is the direct outcome of the velocity aligned activity model, resulting in a promoted correlation between neighbor velocities through their interactions. Linear polymers with this kind of activity demonstrate bistable behavior with “perpendicular” and “stretched” states. The star topology significantly enriches this behavior by breaking the correlation between the arms at the central bead, thus effectively generating the combinatorial number of states in the system (four states for the three-armed star above, and more for a greater number of arms). In contrast, ABP star polymers (see Figure 4 below) are known to stretch gradually at increasing activity with no particular ordering of the system.

The influence of the number of arms on the molecule size in a star polymer in relation to a linear polymer can be characterized by a parameter g(n) defined as $g\left(n\right)=\langle R_{g,n}^{2}\rangle /\langle R_{g,1}^{2}\rangle$, where $\langle R_{g,n}^{2}\rangle$ is a squared gyration radius of a star polymer with the number of arms *n*, and $\langle R_{g,1}^{2}\rangle$ is the squared gyration radius for a linear chain with the same number of monomers. For passive star polymers, this quantity has been computed by Kalyuzhnyi et al. with dissipative particle dynamics (DPD) simulations and analytical theory [48]. Shown in Figure 3d is g(n) for the active stars with up to 6 arms. The slope at the lower activity-to-temperature ratio is consistent with the results of [48] (taking into account a modest number of the arms in our case, which does not allow one to reach any theoretical scaling). The slope for the stretched stars is much steeper, highlighting again the substantial change in structural behavior above the stretching transition.

### 2.2. Structure of Stars Made of Active Brownian Particles

In comparison to the correlated activity results shown in the previous subsection, the uncorrelated one produces a substantially more simplistic picture. Figure 4 shows the main structural characteristics for star-shaped polymers in the ABP model as a function of the number *n* and length *N* of arms. Contrary to the behavior in Figure 3a, $R_{g}$ for ABP shows scaling ≈3/5 for all activities. The little contraction at small $f_{a}/\left(k_{B}T\right)$ found for linear ABP chains disappears for stars as a consequence of additional excluded volume interactions between the different arms, which prevent the reduction of $R_{g}$ (inset in Figure 4c). More importantly, the stretching with growing activity for the ABP model does not exhibit a steep extension at intermediate activities (Figure 4c), and the swelling occurs gradually without coexisting steady states (Figure 4b), as was the case for the velocity-aligned model. In all these cases, the qualitative behavior of an ABP active star is the same as for an ABP active linear chain. Still, the difference between linear and star polymers persists in the parameter g(n) (Figure 4d). It is seen that, as the number of arms increases, the chain size increasingly diverges from the size of a linear chain with the same number of monomer units, which is obviously also characteristic of passive star polymers. At very high activity, however, a slight change in the slope of the form factor g(n) versus the number of arms is observed. Again, this is consistent with the passive star results in [48], with the remark that, with excluded volume interactions in play, one cannot properly simulate too many arms and reach the theoretical scaling. Altogether, one can infer that the topology makes a difference only if the activity is correlated.

### 2.3. Dynamical Multistability in Velocity Aligned Active Stars

**Figure 5:**
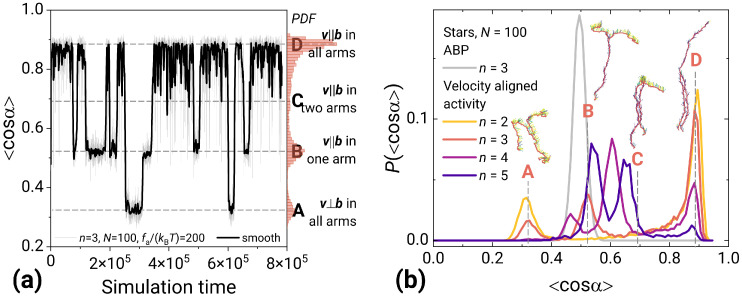
Order parameter <cosα> for the stars made of velocity-aligned activity monomers. (**a**) Evolution of <cosα> during simulation time for the star with three arms, N=100, $f_{a}/\left(k_{B}T\right)=200$. On the right side, the corresponding probability distribution of <cosα>. Dashed lines show the peaks of distribution, corresponding to the particular conformations of the star, from A to D, respectively. (**b**) Distribution of <cosα> for the stars with N=100 and a different number of arms *n*. The snapshots of n=3 star show the steady states corresponding to peaks marked with the dashed lines. For the reference, the distribution for an ABP star is added in grey.

From the structural characteristics, it is clear that the correlated activity is a game changer. In terms of dynamics for the velocity aligned model both the active stars and the linear polymers have two or more preferential orientation configurations for the chain movement. We compute the order parameter <cosα> for a given system snapshot as an average of the cosine of the angle between the velocity of a monomer and the bonds to its neighbors (Figure 5). Along the trajectory, this order parameter switches between two states for a linear chain and n+1 states for a star polymer with *n* arms. For a linear chain, the more likely state corresponds to the instant velocities of beads being oriented almost perpendicular to the chain. The other state corresponds to the instant velocities being oriented along the chain. With more arms added to the picture, the states correspond to the cases of some arms being oriented perpendicular and some along the macroscopic movement of the polymer (Figure 2 and Figure 5). The time-averaged distribution of the order parameter also shows the states clearly (Figure 5b; see also Appendix A, where it is shown that the steady state is reached). The arms switch between the states more or less independently, which tells us that the crosslinker basically breaks the correlation. In experiments for an active chain of connected monomers made of emulsion droplets [29], it was observed that, after a while, these chains start to move perpendicularly to a chain itself consistent with our simulations for the correlated active polymer chains. The authors explain this effect with the impact of chemo-hydrodynamic self-interactions that induce the correlations into the movement of their free-jointed chain of droplets. For the uncorrelated activity, i.e., the ABP polymer, no orientation is found (Figure 2 and Figure 5b). The order parameter just fluctuates around 0.5. This underlines the importance of the correlations in activity. In terms of the mean-squared displacement of the center of mass for both models, we observe a relatively trivial and typical active matter picture when a ballistic motion happens at short times and the Brownian one at long times.

## 3. Simulation Models and Methods

### 3.1. The Interaction Potentials and the Simulation Procedure

The bonding between connected monomers was modeled with a harmonic potential $U_{b}=-1/2K_{B}{\left(l-l_{0}\right)}^{2}$, where $K_{B}=10^{4}$ is the spring constant, *l* is a bond length, and $l_{0}=1$ is a bond equilibrium length. Hereafter, all parameters are given in dimensionless units. Non-bonded monomer units interacted additionally with Weeks–Chandler–Andersen (WCA) potential, which excluded volume interactions, $U_{\mathrm{WCA}}=4\epsilon \left[{\left(\sigma /r\right)}^{12}-{\left(\sigma /r\right)}^{6}\right]$, with standard parameters ϵ=1, σ=1. All monomers had a unit mass.

We performed underdamped Langevin dynamics simulations corresponding to the equation of motion

(1) $$m\frac{d^{2}r_{i}}{dt^{2}}+\gamma \frac{dr_{i}}{dt}=-\nabla U\left(r\right)+F_{a}^{i}+F_{r}^{i},$$

where γ=1 is the friction coefficient, U(r) is the sum of the bond and WCA potentials, $F_{a}^{i}$ is the active force applied to the *i*-th monomer (see the details below), and $F_{r}^{i}$ is a random force associated with implicit solvent at a temperature *T*, obeying the fluctuation-dissipation theorem $\langle F_{r}^{i}\left(t\right)\cdot F_{r}^{j}\left(t^{\prime }\right)\rangle =6k_{B}T\gamma \delta_{ij}\delta \left(t-t^{\prime }\right)$.

We used LAMMPS molecular simulation package [62] with the Velocity–Verlet integration scheme with a time step varied from 5 ×$10^{-4}$ to $10^{-3}$, depending on the simulation setup. Simulation runs were in the range from 5 ×$10^{8}$ to 2 ×$10^{9}$ timesteps. For each set of parameters, 3–10 independent runs were performed.

### 3.2. Monomer Activity Models

**The active Brownian particles model**. In the ABP model, each monomer has its independent intrinsic active force orientation vector $n_{i}$. The corresponding active force is equal to $F_{a}^{i}=f_{a}n_{i}$, where $f_{a}$ is an amplitude of the active force, considered the same for all monomers (Figure 1b). The orientation vector $n_{i}$ is driven by the rotational Langevin equation $\gamma_{r}dn_{i}/dt=\eta_{i}\times n_{i}$. Here $\eta_{i}$ is a torque noise with zero mean, variance $\langle \eta \left(t\right)⊗\eta \left(t^{\prime }\right)\rangle ={\left(2k_{B}T\right)}^{2}\delta \left(t-t^{\prime }\right)/D_{r}$, and $\gamma_{r}=k_{B}T/D_{r}$ is the rotational friction coefficient. $D_{r}$ is the rotational diffusion coefficient linked to the translational diffusion coefficient *D* as $D_{r}=3D/l_{0}^{2}$.

**The velocity-aligned activity model**. In this model, active force acting on an individual monomer is aligned with its instantaneous velocity, $F_{a}^{i}=f_{a}v_{i}/\left|v_{i}\right|$, where $f_{a}$ is an amplitude of an active force.

For both models, we varied the ratio between activity and diffusivity of the system $f_{a}/\left(k_{B}T\right)$ in the range from 0 (passive) to 300 (highly active) by changing the temperature $k_{B}T$ from 0.0033 to 1, respectively, at a fixed $f_{a}=1$. For the case of fully passive polymer $f_{a}/\left(k_{B}T\right)$ = 0, we set $k_{B}T$ = 1 and $f_{a}=0$.

### 3.3. On the Choice of the Activity Parameters

As observed before [25], in the velocity aligned model, the behavior of even linear active chains does not depend solely on the ratio of two characteristic scales, the thermal motion scale set by *T*, and the activity scale defined by $f_{a}$. This ratio enters a dimensionless parameter known as Péclet number. For a single ABP, the Péclet number reads $Pe=f_{a}\sigma /\left(k_{B}T\right)$, where $k_{B}$ is the Boltzmann constant, and σ is a typical particle size. However, in systems where the active agents interact or are correlated, these or alternative dimensionless parameters may not catch the whole picture [23,40]. For instance, the real MD simulations involve a friction via a viscosity parameter that does influence the results. Furthermore, the activity correlations that are necessarily introduced through the bonds or interactions between the beads (including the case of Vicsek alignment) are not accounted for. Hence, the Pe number is not a single universal parameter, and we refrain ourselves from using it in our plots preferring the explicit $f_{a}/\left(k_{B}T\right)$ quantity.

## 4. Conclusions and Final Remarks

To summarize, we have assessed how the correlation in activity affects the structural and dynamical behavior of polymers with the simplest non-trivial topology in the form of a star architecture and compared it with the linear case. The correlations in activity produce collective effects leading to a co-existence of different states with various degree of stretching along the same simulation trajectory. The correlations also induce the collective dynamics and ordering of the whole chain or some of the arms perpendicular to or along the velocity. The perpendicular ordering is qualitatively consistent with available experimental data for the dynamics of active oligomers made of connected emulsion droplets [29]. The uncorrelated activity of the ABP model fails to produce the effects described above. Our results illustrate that non-trivial effects could be a consequence of this correlated activity. Thus, a further and more thorough development of active polymer models taking into account more complex collective effects is needed.

One could also compare velocity aligned model results with the effects of models with an activity interplay with correlations in bond directions or in simple words with an effect of chain rigidity. For semiflexible active linear polymers [7,8], no abrupt extension is observed at intermediate activities. Moreover, the higher activity increases the bending and leads to an intermediate shrinkage of a molecules. No co-existing metastable states were found in that case. Thus, the corresponding correlations in the models produce quite a different effect on the behavior.

While branching is one of the options of departure from a linear topology, another alternative is connecting the ends to form a ring. The ring active polymers were studied both analytically and in simulations [18,34,63,64]. A size-dependent collapse and arrest of active ring polymers was investigated by Emanuele et al. [18], where an active self-avoiding ring polymer was constructed by self-propelled monomers. Crazing of non-concatenated ring polymers in a glassy state [64] and ring polymers behaving differently than linear polymers in a mixture of active–passive polymer mixtures [63] have also been reported. A very recent preprint also discusses the influence of branching for passive polymers in a bath of active particles [65]. These non-equilibrium type environments are known to affect the passive polymer behavior [66]. This additionally supports the fact that topology is important in active systems.

## Figures and Tables

**Figure A1:**
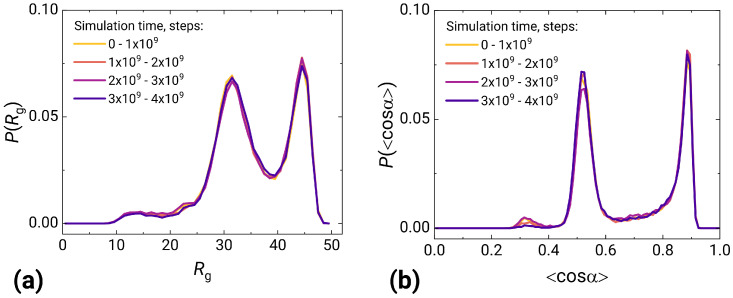
Comparison of (**a**) the probability distribution functions for the gyration radii and (**b**) the order parameters <cosα> obtained from 4 different parts of a simulation trajectory for n=3, $N=100,f_{a}/\left(k_{B}T\right)=200$ in the case of the velocity-aligned model.

## Data Availability

The raw data supporting the conclusions of this article will be made available by the authors on request.

## Appendix A. Stability of Coexisting Conformation States Within the Transition Region

In Figure A1, we show that our trajectories describe a steady state with a few coexisting conformations of active star polymers. A long simulation trajectory is split into 4 parts. For every part, the probability distribution functions for the gyration radii and (b) the order parameters <cosα> are produced and then plotted together. Since the curves coincide, one can see that the system has reached the steady state.
